# Cerebral Ischemic Preconditioning Aggravates Death of Oligodendrocytes

**DOI:** 10.3390/biom12121872

**Published:** 2022-12-14

**Authors:** Teng Guan, Ying Guo, Chengren Li, Ting Zhou, Qiang Yu, Chaoxian Yang, Guohui Zhang, Jiming Kong

**Affiliations:** 1 Department of Human Anatomy and Cell Science, University of Manitoba, Winnipeg, MB R3E 0J9, Canada; 2 Department of Forensic Medicine, Hebei North University, Zhangjiakou 075000, China; 3 Department of Obstetrics and Gynecology, Guiqian International General Hospital, Guiyang 550024, China; 4 The Second Affiliated Hospital of Chongqing Medical University, Chongqing 400010, China; 5 Department of Anatomy and Histoembryology, School of Basic Medical Science, Southwest Medical University, Luzhou 646099, China

**Keywords:** neurodegeneration, preconditioning, white matter injury, ischemic stroke, oligodendrocytes

## Abstract

Neurodegeneration can benefit from ischemic preconditioning, a natural adaptive reaction to sublethal noxious stimuli. Although there is growing interest in advancing preconditioning to preserve brain function, preconditioning is not yet considered readily achievable in clinical settings. One of the most challenging issues is that there is no fine line between preconditioning stimuli and lethal stimuli. Here, we show deleterious effect of preconditioning on oligodendrocyte precursor cells (OPCs). We identified Bcl-2/adenovirus E1B 19-kDa interacting protein 3 (BNIP3), a mitochondrial BH3-only protein specifically involved in OPCs loss after preconditioning. Repeated ischemia stabilized BNIP3 and increased the vulnerability of OPCs to subsequent ischemic events. BNIP3 became mitochondrial-bound and was concurrent with the dysfunction of monocarboxylate transporter 1 (MCT1). Inhibition of BNIP3 by RNAi or necrostatin-1 (Nec-1) and knocking out of BNIP3 almost completely prevented OPCs loss and preserved white matter integrity. Together, our results suggest that the unfavorable effect of BNIP3 on OPCs should be noted for safe development of ischemic tolerance. BNIP3 inhibition appears to be a complementary approach to improve the efficacy of preconditioning for ischemic stroke.

## 1. Introduction

Since the initial description by Murry et al. on cardioprotection, ischemic preconditioning is well documented as an adaptive reaction that provides tolerance to ischemic damage through transient sublethal stimuli [1]. Preconditioning typically results in augmented gene expression and cellular metabolism. Insights on preconditioning also translate to cerebral ischemia. Preconditioning-induced neuroprotection is widely observed although its effects are often complicated due to the cell type-specific reactions in the central nervous system [2,3]. Neurons gain tolerance in ischemic preconditioning through alterations in the ability of mitochondria and endoplasmic reticulum (ER) to manage calcium homeostasis [4]; Astrocytes, on the other hand, provide bioenergetic lactate to neurons in preconditioning [5]. With their glycogen content, astrocytes are capable of preserving metabolic glycolysis and glycogenolysis, supporting neuronal activities, and modulating glutamate uptake to prevent further excitotoxicity. While research on microglia focuses on M2 polarization and upregulation of remodeling factors as a therapeutic strategy. The response of oligodendrocytes (OLs) to ischemic preconditioning is largely unknown [6].

OLs are glial cells that differentiate from oligodendrocyte progenitor cells (OPCs). Apart from their myelinating function, OLs provide metabolic support to neurons [7]. Impaired blood flow damages the communication between OLs and axons leading to functional disability and cognitive deficits [8]. Although stroke survivors may achieve a full physical recovery, their emotional and behavioral abnormalities can become persistent, severe, and distressing, suggesting that promoting myelination might yield improved clinical outcomes [9].

The OPCs present in substantial numbers in the adult central nervous system (CNS) and are relatively quiescent but retain regenerative capacities to myelinate axons [10]. OPCs and late OL progenitors are more susceptible to ischemic injury than neurons, astrocytes, or microglia [11]. Unlike astrocytes, OL lineage cells do not have glycogen as a form of energy storage, and a decline in energy production may rapidly cause mitochondria malfunction and delay lipid synthesis in OPCs [12]. Energy deprivation activates alpha-amino-3-hydroxy-5-methylisoxazole-4-propionic acid, kainate, and N-methyl-d-aspartate glutamate receptor types and contributes to excitatory injury [13]. In addition, the microglia are activated to the M1 phenotype by ischemic injury and trigger the release of inflammatory cytokines, which can negatively affect glutamate uptake by astrocytes and exacerbate the excitotoxicity of OPCs. With disease progression, remyelination may eventually fail due to the exhaustion of OPCs.

It’s known that stroke patients with a history of transient ischemic attacks (TIA) experience less brain damage than stroke patients without any history of TIA [14]. The mechanism underlying such tolerance has been associated with preconditioning. However, TIA also increases the risk for post-traumatic stress disorder (PTSD) and is associated with anxiety/depression, implicating the involvement of oligodendrocyte lineages in such psychiatric disorders [15]. These findings prompted us to explore whether preconditioning is insufficient because it may not adequately precondition all the cell types in the brain.

## 2. Materials and Methods

### 2.1. Animals

All experiments were done following the guidelines approved by University of Manitoba Institutional Animal Care and Use Committee. For in vivo study, all mice were adult males, 12~14 weeks old. Wide-type (WT, B6; 129) and BNIP3 knockout mice (B6; 129-BNIP3^tm1Gwd^) were obtained from Dr. Spencer Gibson’s lab in Cancer Care Manitoba. For in vitro study, one-day-old neonatal Sprague Dawley rats and C57BL/6 mice were obtained from Central Animal Care Services at the University of Manitoba. WT and BNIP3 knockout mouse pups were also used for in vitro assay. Animals were housed under temperature-controlled conditions with a 12 h light/dark cycle and ad libitum access to water and food. All efforts were made to minimize animal suffering and reduce the number of animals used.

### 2.2. Middle Cerebral Artery Occlusion

Transient focal cerebral ischemia was induced using an intraluminal filament model of middle cerebral artery occlusion (MCAO), as described previously [16]. Briefly, mice were anesthetized with isoflurane (1.5–2%), and rectal temperature was maintained at ~37 °C. A 6-0 silicone rubber-coated nylon monofilament (Doccol Corp, Sharon, MA, USA) was inserted via the right external carotid artery until it obstructed the MCA. Mice with >85% flow reductions during the ischemic period were included in this study. For the sham MCAO surgery, vessels were visualized and cleared from connective tissue, but the MCA was not occluded. After surgery, animals were allowed to recover in a warmed chamber before being returned to their home cages.

### 2.3. Measurements of Infarct Volume

Mice were anesthetized by isoflurane overdose. Upon cessation of reflexes, mice were transcardially perfused with saline, and the brains were dissected immediately after decapitation. Brains were cut into equally spaced (2 mm) coronal blocks for Western blot assay. For 2,3,5-triphenyl tetrazolium chloride (TTC) staining, coronal brain tissue slices (2 mm in thickness) were stained for 15 min at 37 °C with 2% TTC (Sigma, St. Louis, MO, USA) in phosphate-buffered saline (PBS, 137 mM NaCl, 2.7 mM KCl, 8 mM Na_2_HPO_4_, and 2 mM KH_2_PO_4_) solution. The Infarct area was determined with ImageJ software and volume was calculated by multiplying the thickness of the brain section. To minimize the error introduced by edema, infarct volume was obtained as follows: corrected infarct volume = contralateral hemispheric volume − ipsilateral non-infarcted volume.

### 2.4. Primary Cell Culture

OPCs were differentiated from the subventricular zone (SVZ) sphere of wild-type (WT) and BNIP3 knockout (KO) mice as described by Chen et al. [17]. WT and BNIP3 KO mice (8–12 weeks old) were deeply anesthetized with isoflurane and then euthanized by decapitation. The SVZ was dissected, and the dissociated SVZ single cells were re-suspended in a proliferation medium consisting of Neurobasal Medium (Gibco, Billings, MT, USA), B27 neuronal supplement (Gibco), and 20 ng/mL epidermal growth factor (EGF, PeproTech, Cranbury, NJ, USA)/fibroblast growth factor-2 (bFGF, PeproTech). Around the fourth day after the formation of SVZ spheres, the culture medium was gradually changed to 20 ng/mL platelet-derived growth factor-AA (PDGF-AA, PeproTech)/bFGF-containing oligosphere medium. After 10 days of induction, the oligospheres were enzymatically dissociated, collected, and plated at the density of 1 × 10^4^ cells/cm^2^ into T-25 culture flasks or 24-well plates with 12-mm-diameter glass coverslips for experiments.

For some experiments, rat postnatal OPCs were obtained as described according to the original procedure of McCarthy and de Vellis [18]. Postnatal one-day rat pups were sacrificed, and cerebral cortices were dissected and mechanically dissociated. Mixed cultures were then grown in Dulbecco’s Modified Eagle’s Medium (DMEM, HyClone, Logan, UT, USA) supplemented with 20% fetal bovine serum (Gibco). At 10 days in vitro, flasks containing the cultures were shaken at 220 rpm for 1 h to remove microglia. The cultures were then shaken at 220 rpm overnight to dislodge the loosely attached OPCs. OPCs were further purified from astrocytes and microglia by being placed in uncoated tissue culture dishes for 1 h and then gently swirled. The non-adherent cells were collected and seeded at the density of 1 × 10^4^ cells/cm^2^ into T-25 culture flasks or 24-well plates with 12-mm-diameter glass coverslips for experiments.

### 2.5. Lentivirus Transfection, Oxygen-Glucose Deprivation (OGD), and Pharmacological Treatment

The shRNA sequences were designed using Invitrogen’s BLOCK-iT RNAi Designer. The oligonucleotides were synthesized, annealed to generate double-stranded oligos, and cloned to pENTR/U6 vectors (Invitrogen, Waltham, MA, USA). After the shRNAs were co-transfected with BNIP3 plasmids into HEK293T cells, the inhibition efficiencies of the shRNAs were determined by immunofluorescence microscopy and quantitative Western blot. Selected shRNA sequences were inserted into Invitrogen’s BLOCK-IT Lentiviral RNAi Expression system. Lentiviral stocks were produced using ViraPower Packaging Mix (Invitrogen). Transduction was performed by adding lentiviral stocks into each well 24 h before OGD treatment. For hypoxia treatment, cultures were incubated with glucose-free Earle’s Balanced Salt Solution (EBSS, 117 mM NaCl, 5.3 mM KCl, 26.2 mM NaHCO_3_, 1.8 mM CaCl_2_, 1 mM NaH_2_PO_4_·H_2_O, 0.8 mM MgSO_4_) placed in a modular incubator chamber and infused with mixed gas (95% N_2_ and 5% CO_2_) at 37 °C·until a specified time point (0.5 h as preconditioning followed by 0.5, 1, 2 h OGD treatment, respectively) was reached. All cultures were then switched back to the normal culture medium and returned to a normoxic incubator. Necrostatin-1 (Nec-1) was immediately applied after cells were exposed to OGD.

### 2.6. Cell Counts and Viability Assay

Cell counts were performed to determine the cell viability, and images were obtained with a 20× objective on a fluorescent microscope (ZEISS, Jena, Germany). Border rules were used, and cell counts were obtained followed by manual verification. Viable cell counts were presented as a percentage of the total cell number. Cell viability was also measured using Premix WST-1 Cell Proliferation Assay kit (Clontech, San Jose, CA, USA). Four hours prior to the end of the experiment, WST-1 premix solution was added to the wells and mixed carefully. Formazan dye formed by metabolically active cells was quantified by measuring its absorbance height at 440 nm using a microplate reader.

### 2.7. Western Blots

Protein lysates samples were loaded on 12% SDS polyacrylamide gel and then transferred to a polyvinylidene difluoride membrane. Membranes were blocked with 5% (*w*/*v*) milk in TBS buffer containing 0.05% Tween-20 for 1 h and then incubated with a mouse monoclonal anti-BNIP3 (1:1000; provided by Dr. A. Greenberg) [19] antibody overnight at room temperature. Blots were washed with TBS buffer and then incubated with peroxidase-labeled secondary antibodies for 2 h at room temperature. Peroxidase activity was visualized with the Enhanced Chemiluminescent Substrate (PerkinElmer, Waltham, MA, USA) according to the manufacturer’s instructions. The optical density of the specific band was normalized based on the loading controls. Chemiluminescent signals were captured on autoradiography and were used to assess protein content.

### 2.8. Immunofluorescent Staining, TUNEL, and Microscopy

Frozen coronal sections (14 μm) of the brains were sliced after perfusion and post-fixed with 4% paraformaldehyde in PBS at 4 °C overnight. Cell cultures on glass coverslips were fixed for 30 min in 4% paraformaldehyde. Brain sections or cell cultures on coverslips were washed three times with PBS, blocked with 1% BSA PBST (137 mM NaCl, 2.7 mM KCl, 8 mM Na_2_HPO_4_, 2 mM KH_2_PO_4_ and 0.25% Triton X-100) for 30 min at room temperature, and then washed with permeabilized PBS containing 0.25% Triton X-100. The cells were incubated with the following primary antibodies: rabbit anti-BNIP3 (Abcam (Cambridge, UK), Ab10433, 1:500), mouse anti-A2B5 (Sigma-Aldrich, MAB312, 1:500), mouse anti-SMI-32 (Sigma-Aldrich, 5.59844, 1:500), mouse anti-NG2 (Sigma-Aldrich, 05-710, 1:500), rabbit anti-MCT1 (Santa Cruz (Santa Cruz, CA, USA), SC-50325, 1:50; Abcam, ab90582, 1:500), rabbit anti-PDGFRα (Santa Cruz, sc-398206, 1:200), goat anti-MBP (Santa Cruz, sc-13914, 1:5000) and goat anti-AIF (Santa Cruz, sc-9416, 1:100) overnight at 4 °C. After being washed three times, the sections and cultures were incubated with Alexa Fluor^®^ 594 goat anti-mouse IgG and Alexa Fluor^®^ 488 goat anti-mouse IgG (Invitrogen, 1:1000) for 2 h at room temperature. After being washed, immune-stained sections and coverslips were incubated with Hoechst 33342 for identification of the nuclei (Sigma, at a final concentration of 1 µg/mL). Negative controls were performed by using PBS instead of the primary antibodies. To detect DNA degradation, the DeadEnd™ Fluorometric TUNEL System (Promega, Madison, WI, USA) was applied following the manufacturer’s instructions. The slides were observed by a Carl Zeiss Axio Imager. Z2 (Carl Zeiss, Gottingen, Germany). Images were taken AxioCam MRm (Carl Zeiss, Gottingen, Germany) and were processed with the ZEN software (blue edition, 2.3, Carl Zeiss). To evaluate the colocalization, Pearson’s correlation coefficient was used as previously described with modification [20]. For immunofluorescence intensity measurements, staining and image acquisition were performed in parallel for the specific set to compare multiple specimens. 6–9 micrographs of each sample under different visual fields were analyzed, and the averaged intensities were represented.

### 2.9. Toluidine Blue Staining and Transmission Electron Microscopy (TEM)

Animals were transcardially perfused with 2% glutaraldehyde/2% paraformaldehyde. The brains were then cut into 1 mm thick sections, and the corpus callosum was manually dissected. Samples were fixed for 1 h in a solution of 2.5% glutaraldehyde that was diluted with 0.1 M Sorensen’s buffer (prepared by mixing 5.24 g NaH_2_PO_4_·H_2_O and 23.0 g Na_2_HPO_4_ with 1000 mL ddH_2_O, Ph = 7.4). Then, the samples were washed by immersion in 0.1 M Sorensen’s buffer containing 5% sucrose at 4°C overnight. The next day, the samples were further washed in 0.1 M Sorensen’s buffer containing 5% sucrose two times for 40 min each at room temperature. Post-fixation was processed by adding 1% O_s_O_4_ to the cell samples for 2 h. After being dehydrated with graded alcohols and methanol, samples were embedded in epoxy resin and then dried in an oven at 60 °C for 24 h. Semi-thin sections were prepared from a resin block using a glass knife and stained with 0.5% (*w*/*v*) toluidine blue solution and examined by light microscopy (Nikon ECLIPSE 80i, Tokyo, Japan). Myelin density was quantified using binary masks by thresholding the images. For TEM, A microtome (Leica, Concord, ON, Canada) was used to obtain thin sections, which were further mounted on copper grids. Finally, 2% uranyl acetate and 1% lead citrate were used to stain the samples. The prepared grids were analyzed with a transmission electron microscope (Philips CM-10, Hillsboro, OR, USA) at 25 °C. G ratios (the ratio of axon diameter to the axon plus myelin sheath diameter) were calculated using the Image J software (1.53 c) for at least 100 fibers per animal.

### 2.10. Statistical Analysis

All statistical analyses were performed using one-way ANOVA and Tukey’s tests. Differences among groups were analyzed using Student’s *t*-test. Differences were considered statistically significant at *p* < 0.05.

## 3. Results

### 3.1. Preconditioning Does Not Prevent Striatal Infarct after Ischemic Stroke

The filament occlusion of the MCA is a well-established method that elevates metabolic stresses in the ischemic penumbra. Occlusion with MCA was confirmed by an 85% drop in cerebral regional blood flow using laser Doppler flowmetry (LDF), which could be restored to normal after suture withdrawal. MCAO for 15, 10, and 3 min as preconditioning and recovery for 1 day were screened based on the principle of not causing damage when applied along (Supplement Figure S1). Preliminary experiments showed that 15- or 10 min MCAO produced a pale TTC staining, an indicator of mitochondrial dysfunction in the ipsilateral striatum at 72 h of reperfusion. When we reduced the duration of preconditioning MCAO to 3 min, striatal infarcts were not observed. Three minutes was selected because it did not produce striatal infarcts and showed comparable protection to those preconditioned with 10 min MCAO. In the sham preconditioning groups, mice underwent the same duration of anesthesia and the same surgical procedures that were performed in the preconditioning group. Twenty-four hours later, mice were subjected to ischemic stroke induced by 1 h MCAO, which was in line with commonly used occlusion time in mice.

**Figure 1:**
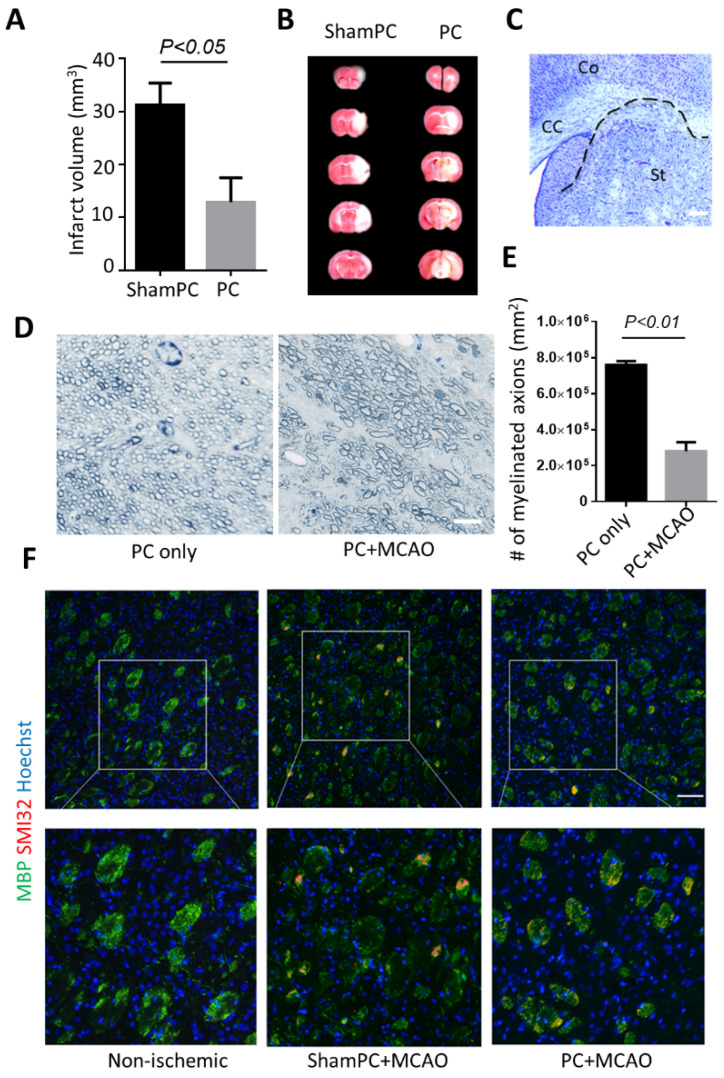
Preconditioning attenuates ischemic injury in the cortex but not the striatum. (**A**) Quantitative analysis of ischemic area based on TTC staining. (**B**) Photomicrographs of infarct areas stained with TTC in shamPC and PC group. ShamPC, mice received sham preconditioning (i.e., without preconditioning treatment); PC, mice received 3 min of MCA occlusion as preconditioning. (**C**) Demonstration of tissue swelling in a representative mouse subcortical white matter 7 days after stroke. CC indicates corpus callosum; Co, cortex; and St, Striatum. (**D**) Toluidine blue staining of cross sections of the corpus callosum. Bar, 5 μm. (**E**) Fewer myelinated axons were observed in PC + MCAO group. (**F**) Representative immunofluorescence microscopic analyses of MBP (green) and SMI-32 (red) in shamPC and PC group after MCAO. MBP with diffuse distribution and abnormally dephosphorylated neurofilament SMI-32 could be detected in both preconditioning/non-preconditioning-treated striatum. Results shown represent mean ± SD, *n* = 5–7. Bar, 100 μm.

There was no mortality after stroke when assessed 7 days after MCAO, and no differences in body temperature were detected between groups during surgery and recovery. The white matter infarctions were observed in most of the animals. With 3 min MCAO preconditioning performed 24 h before 60 min of MCAO, infarct volume in the striatum was significantly decreased (about 60% reduction in total infarct size) compared to the sham preconditioning group (Figure 1A,B). We noticed that a degree of tissue swelling persisted in preconditioning-treated mice (Figure 1C). Myelinated axons counted by toluidine blue staining (Figure 1D) revealed MCAO-induced demyelination in preconditioned mice, while preconditioning alone did not show myelin abnormalities (Figure 1E). We performed further immunohistochemistry staining of myelin basic protein (MBP) and neurofilament H (Non-Phosphorylated, SMI-32) as shown in Figure 1F. As neurofilaments are highly phosphorylated under physiological conditions, changes in the state of phosphorylation can be used to detect axonal injury. Myelin impairment can also be evaluated with immunostaining of MBP. Our data suggested that MBP staining tended to be distributed diffusely throughout the white matter tracks without SMI-32 labeling compared with non-ischemic mice. Intense expression of SMI-32 was detected after MCAO in the fiber fascicles in both MCAO alone and preconditioning-treated striatum. MBP immunofluorescent staining displays a noticeable decrease of myelin in the striatum in MCAO mice, while preconditioning group showed some degree of protection (Figure 1F).

### 3.2. BNIP3 Knockout Mice Have Less Cerebral Damage Compared with Wild-Type Mice

**Figure 2:**
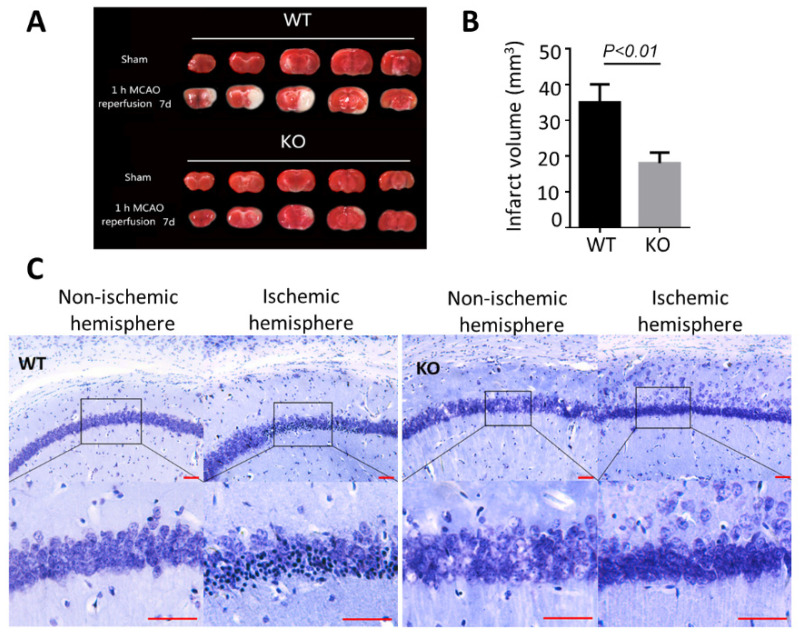
BNIP3 knockout mice have less cerebral damage compared with wild-type mice. (**A**) TTC staining sections of wild-type and BNIP3 knockout 7 days after MCAO. (**B**) Quantitative analysis of ischemic volume based on TTC staining. (**C**) Cresyl Violet Staining (Nissl Staining) staining showed numerous degenerated neurons in CA1 areas. Results shown represent mean ± SD, *n* = 4. Bar, 50 μm.

There were no obvious developmental differences between WT and BNIP3 KO mice at baseline. BNIP3 involves in the activation of caspase-independent apoptosis pathways. We postulated that BNIP3 levels might affect brain injury after ischemia. To determine the severity of the neuronal injury, we first investigated the lesions in areas of the brain that were frequently affected, and the ipsilateral ischemic hemisphere was identified to have a lack of staining in the cortex and striatum. Representative sections from sham-operated and MCAO-induced ischemic brains are presented in Figure 2A. In wild-type animals subjected to 1 h MCAO, brain infarct regions were observed in the ipsilateral corpus callosum, external capsule, and the surrounding gray matter at 7 days of reperfusion after ischemia. BNIP3 knockout remarkably decreased the infarct volume, as shown in Figure 2B. The mutants showed a substantial reduction in infarct volume in the striatum and a moderate reduction in the cortex. We then assessed the hippocampal CA1 sector with Nissl staining 7 days after focal cerebral ischemia (Figure 2C). In the non-ischemic hemisphere CA1, the structure of most neurons in the CA1 sector was clear and uniformly stained. There were extensive neuronal changes in the ischemic hemisphere CA1 sector of the hippocampus in WT MCAO animals. Neurons were shrunk and had deep color staining. At the same time, part of the neurons disappeared, and the rest cell outline was fuzzy. In BNIP3 KO mice, neurons of the CA1 region were largely resistant to ischemic damage, implying knockout of BNIP3 ameliorated neuron degeneration and retained the number of cells in the CA1. Taken together, BNIP3 knockout could not only preserve structure of most neurons but also prevent neuronal loss.

**Figure 3:**
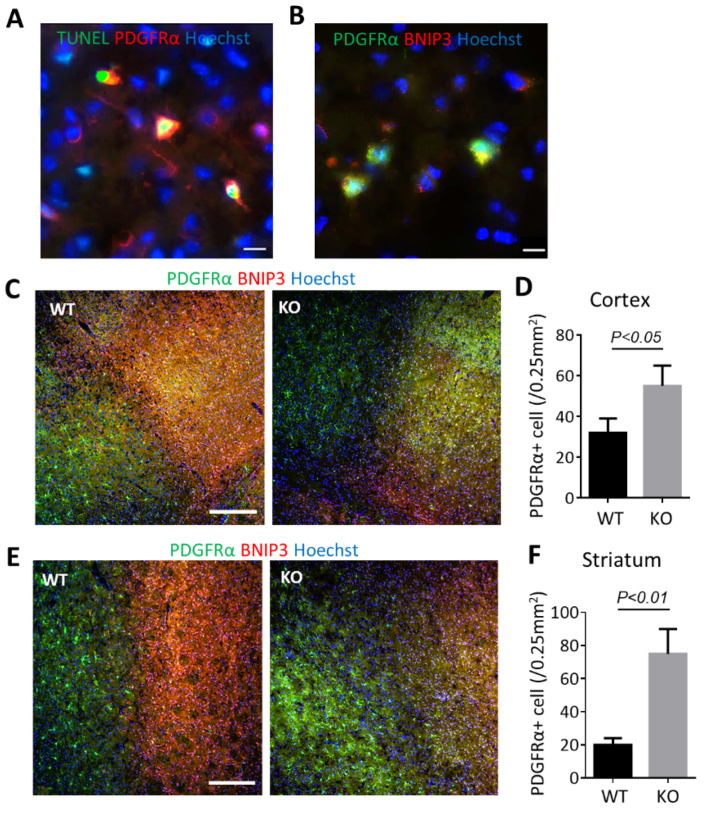
Deficiency of BNIP3 reduces oligodendrocyte death. (**A**) TUNEL assay demonstrated apoptotic cells in ischemic brain were co-labeled with PDGFRα. Bar, 10 μm. (**B**) Double immunohistochemical staining demonstrated PDGFR-α cells were co-labeled with BNIP3. Images were taken from the external capsule (EC) region. Bar, 10 μm. (**C**) Double immunohistochemical staining of PDGFR-α and BNIP3 in the ischemic cortex at 7 days after MCAO. Bar, 100 μm. (**D**) Quantification showed the number of PDGFR-α positive OPCs was significantly higher in the cortex of BNIP3 KO mice than in the wild-type mice. (**E**) Double immunohistochemical staining of PDGFR-α and BNIP3 in the ischemic cortex at 7 days after MCAO. Bar, 100 μm (**F**) Quantification showed the number of PDGFR-α positive OPCs was significantly higher in the striatum of BNIP3 KO mice than in the wild-type mice. Results shown represent mean ± SD, *n* = 4.

To test whether the OPCs were influenced by ischemia, we analyzed TUNEL-positive cells in platelet-derived growth factor receptor (PDGFRα) positive specimens (Figure 3A). The immunostaining images displayed the apoptotic cells (TUNEL positive) were predominantly PDGFRα positive, and most of the PDGFRα positive OPCs were positive with BNIP3 (Figure 3B). Figure 3C,E presented the consistent pan-necrotic lesion involving both cortical and striatal regions of the ipsilateral hemisphere, characterized by the destruction of OPCs and intensive staining of BNIP3 in the wild-type ischemic core. WT mice showed large cortical and subcortical infarcts, and a transition zone between the necrotic area and the healthy tissue was visible. In contrast, BNIP3 KO showed less damage, and the boundaries between the ischemic core and the normal region were broader, displaying a smooth transition. Significant amounts of BNIP3-positive cells were present in the ischemic core only in WT animals, while lots of the BNIP3-expressing cells were co-labeled with PDGFRα (Figure 3C,E). Cortical and striatal ischemic boundary zones were analyzed by quantifying the OPCs population in both regions. PDGFRα-positive cell counts are shown in Figure 3D,F. Altogether, BNIP3 KO significantly increased the number of PDGFRα-positive cells compared with wild-type mice.

### 3.3. BNIP3 Knockout Rescues Myelination Defects after Preconditioning

**Figure 4:**
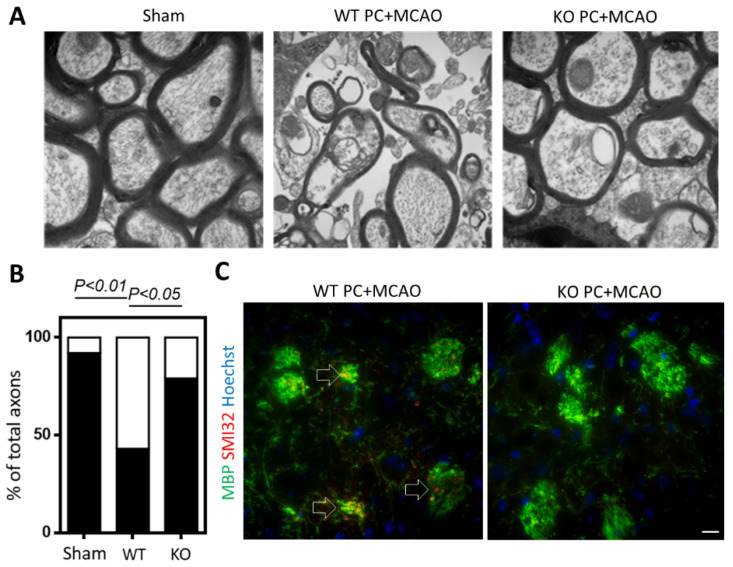
BNIP3 knockout rescues myelination defects after preconditioning. (**A**) Electron micrographs of myelinated axons in the infarcted corpus callosum. EM showed healthy myelinated axons in the control and damaged axons of varying size in the wild-type and BNIP3 KO mice. Note that more vacuolization and myelin debris could be identified in the wild-type preconditioning. (**B**) The percentages of the myelinated axon (black box) and demyelinated axon (white box) were quantified from electron microscopy. (**C**) Representative immunofluorescence microscopic analyses of MBP (green) and SMI-32 (red) in preconditioning treated wild-type and BNIP3 KO mice. Abnormal MBP distribution and dephosphorylated neurofilament SMI-32 could be detected in preconditioning treated wild-type mice. *n* = 3.

To determine whether BNIP3 knockout can rescue myelination defects after stroke, myelin thickness was measured in the corpus callosum using TEM. We applied the same paradigm of preconditioning to the BNIP3 knockout mice, and the result showed that the combination of BNIP3 KO and preconditioning improved myelin ultrastructure. As shown in Figure 4A, the white matter contained compact bundles of myelinated axons with a well-defined septum in the sham-operated group. Axons in the corpus callosum from wild-type animals were still swollen and appeared distorted. The myelinated axons were less compact, vacuoles and myelin debris could be frequently observed. Significant increases in the thick myelinated axon (G-ratio < 0.8) and decreases in the demyelinated axon (0.8 < G-ratio < 1) were observed in BNIP3 KO mice (Figure 4B), suggesting that BNIP3 KO may maintain axonal myelination after preconditioning and stroke. We further addressed the effect of BNIP3 KO on neurodegeneration by labelling non-phosphorylated neurofilament H with SMI32 antibody, which was almost blocked by knocking down of BNIP3, as shown in Figure 4C.

### 3.4. BNIP3 Is Associated with OPC Vulnerability in Preconditioning

**Figure 5:**
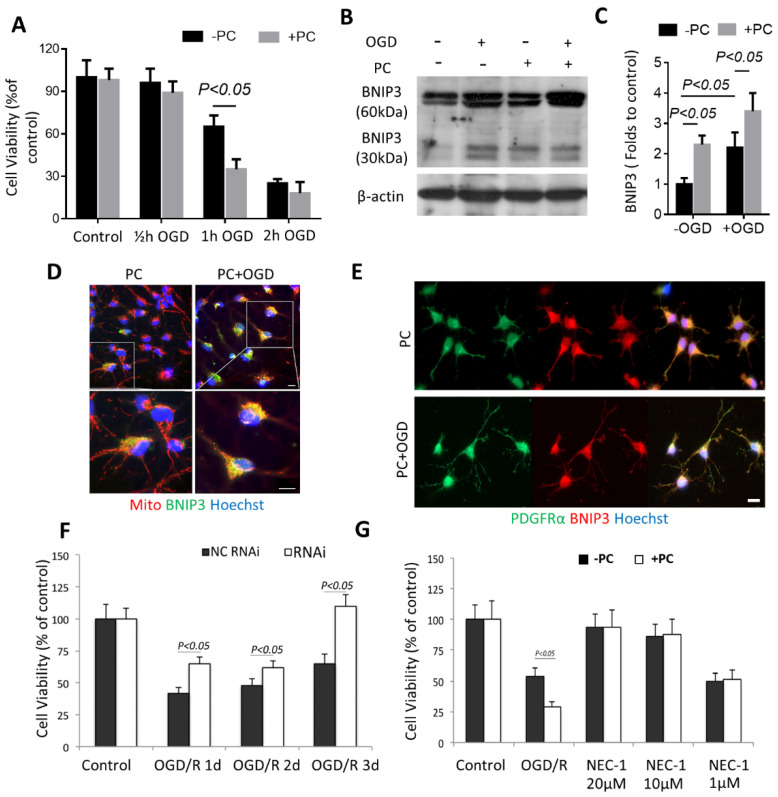
Preconditioning aggravates death of OPCs. The cultured cells were treated with or without preconditioning, and 24 h later, re-exposed to OGD. (**A**) Viability was determined by WST-1 assay. (**B**) Representative Western blots showing expression of BNIP3. (**C**) Densitometric quantification showed the ratio of BNIP3 to β-actin, normalizing the ratio of control as one. (**D**) Immunofluorescence microscopic analyses showed that both preconditioning only and preconditioning + OGD cells had overlapping BNIP3 staining (green) and mitochondria staining (red). Bar, 10 μm. (**E**) Immunofluorescence microscopic analyses showed that both preconditioning only and preconditioning + OGD cells had overexpressing BNIP3 (Red). The Red fluorescence from preconditioning only was weaker and more diffusive compared to preconditioning + OGD cells. Bar, 10 μm. (**F**) Effect of BNIP3 knockdown on OPCs survival. OPCs were infected with BNIP3-shRNA (RNAi) or control scrambled shRNA (NC-RNAi), treated with OGD, and then allowed to recover. Cell viability assays were performed at different time points of reoxygenation. (**G**) Effect of BNIP3 inhibition on OPCs survival, after the lethal OGD, or preconditioned OPCs, either remained untreated or treated with Nec-1 (20, 10, or 1 μM). Viability was determined by WST-1 assay. Results shown represent mean ± SD from 3 independent experiments.

For the in vitro experiments, we first performed a time-course study to determine what duration of OGD exposure would be sublethal. Half an hour, 1 h, and 2 h of OGD were tested. After the OGD treatment, the cultures were returned to glucose-containing media and then transferred to a normoxic incubator, which allowed for reoxygenation. Twenty-four hours later, these cells were used for cell viability assays. As shown in Figure 5A, 0.5 h of OGD exposure did not reduce OPC viability significantly, whereas 1 h of OGD started to show cytotoxicity. Two hours of OGD exposure killed nearly 80% of the cells. Seeing as 0.5 h of OGD exposure did not show cytotoxicity (and therefore was a “sublethal” injury to the OPCs), this initial 0.5 h of OGD was considered “preconditioning.” One hour of OGD exposure, which produced moderate injury to OPCs (~63% OPC survival), was considered “lethal OGD.” We then tested the post-effect of preconditioning on OPCs viability. 24 h after preconditioning, the cultures underwent the second episode of OGD. Cell viability assays were then conducted 24 h after reoxygenation. Preconditioned OPC viability was significantly decreased after lethal OGD.

To study the BNIP3 expression profile in OPCs after lethal OGD, we harvested the cell cultures and quantified BNIP3 protein expressions by Western blot. In contrast to Zhang et al.’s results [21], which show that BNIP3 triggers neuron death in a delayed manner. In our study, after OPCs were exposed to OGD and reoxygenation for 24 h, BNIP3 started to accumulate (Figure 5B, mainly 60 kDa form, faint 30 kDa form). Interestingly, preconditioning itself significantly upregulated BNIP3 expression, and it was confirmed by Western blot (Figure 5C). Preconditioning alone or OGD treatment showed an increased colocalization of BNIP3 and mitochondria by double-staining of BNIP3 and Mito Tracker Red (Figure 5D). After preconditioning, BNIP3 had an intermediate distribution: partially diffuse and co-localized with mitochondria, with a coefficient of 0.71 by Pearson’s correlation coefficient. BNIP3 became mitochondria-bound (Pearson’s R = 0.93) and punctated after lethal OGD (Figure 5D). Thus, patterns of BNIP3 expression and subcellular localization suggest as the amount of OGD exposure increases, BNIP3 expression in OPCs will be increased accordingly (Figure 5E). Once the upregulation of BNIP3 is triggered, another episode of ischemic injury can be the cause of the downstream event. We applied the N167 lentiviral vector to knock down BNIP3 in OPCs. Infection of primary OPCs with the N167 lentiviral vector resulted in almost complete inhibition of BNIP3 in OPCs. A scrambled sequence (S167) was used as the control. It was found that the N167 vector’s inhibition of BNIP3 expression significantly reduced OGD-induced cell death by using a cell viability assay, as shown in Figure 5F. Nec-1 markedly protected the cells after the lethal OGD at a dose of 20 μM or 10 μM of Nec-1, as shown in Figure 5G. Previously, Nec-1 has been shown to prevent BNIP3 expression and block BNIP3-dependent mitochondrial perturbations [22]. Surprisingly, although 1 μM Nec-1 did not reverse OGD-induced injury (i.e., there was no significant difference in cell viability compared to control) but effectively attenuated the preconditioning toxicity (i.e., prevented further cell viability decrease in preconditioned OPCs that had undergone the second episode of OGD). As a result, these data suggest that the initial BNIP3 expression induction is closely related to OPCs susceptibility.

### 3.5. Preconditioning Triggered Oligodendrocytic MCT1 Loss, and the Toxicity to OPC Is Attenuated by Knocking down BNIP3

**Figure 6:**
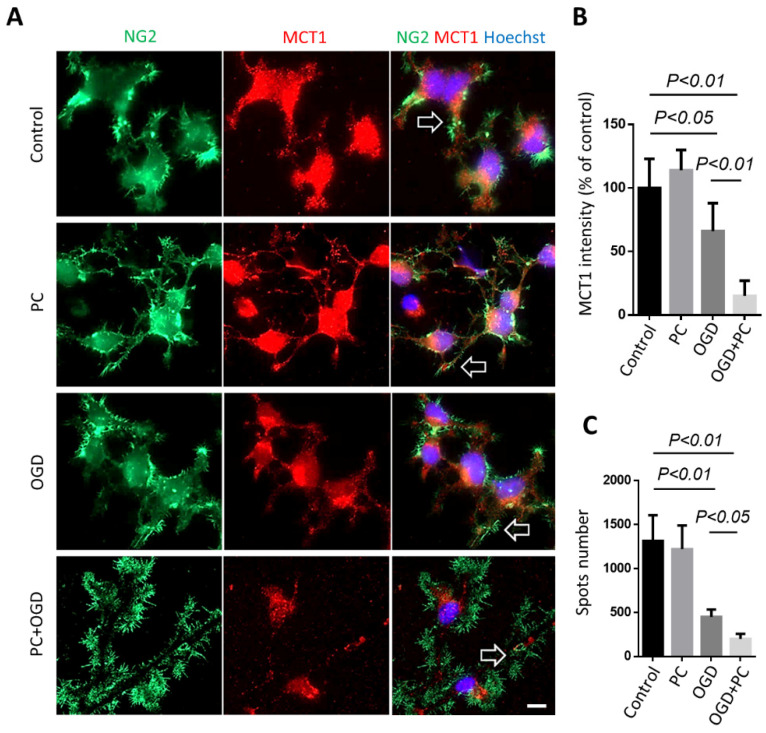
Suppressive effect of lactate utilization of OPCs by preconditioning. (**A**) OPCs were immunostained with NG2 and MCT1 in control, preconditioning only, OGD, and preconditioning + OGD groups, respectively. Signature MCT1 patterns are highlighted with arrows. (**B**) Quantification of relative MCT1 intensity MCT1. (**C**) Quantification of MCT1 spot number on processes in each cell stage. The experiment was performed in triplicate, and 30~50 images were taken for each coverslip. Scale bar = 10 μm.

We found that MCT1 was downregulated with repeated ischemic treatment, although preconditioning (0.5 h of OGD, PC) alone on MCT1 intensity had a slight increase in OPCs (Figure 6A,B). MCT1 was also measured within the cell processes because the lactate transportation is primarily through the end foot membrane, which likely reflects MCT1 transport function. Colocalization coefficient of plasma membrane nerve/glial antigen 2 (NG2) and MCT1 staining showed that MCT1 punctate spots on OPC processes decreased remarkably in PC + OGD cells, but not in PC or OGD cultures, suggesting dysregulation of energy utilization of OPCs after repeated OGD treatment (Figure 6A,C).

**Figure 7:**
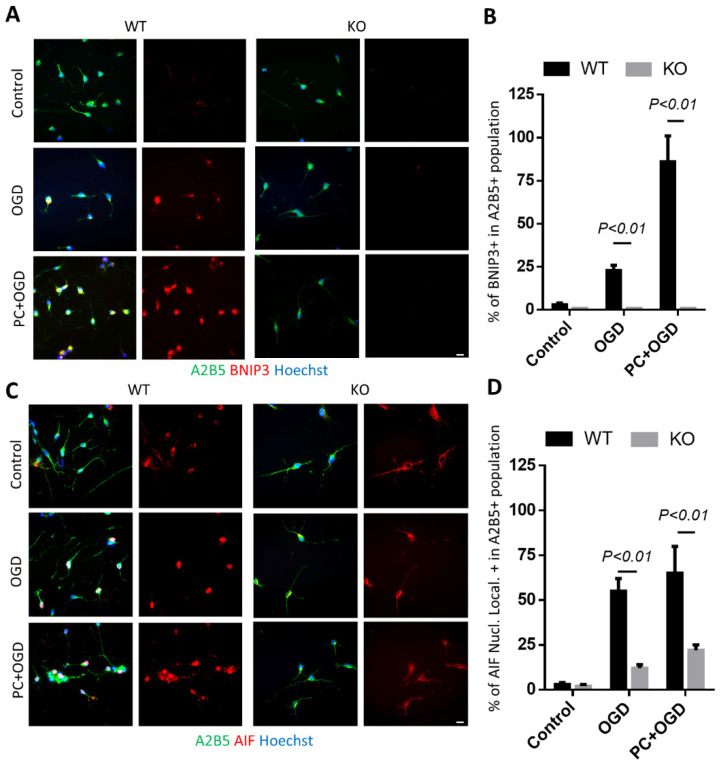
Knockout of BNIP3 ameliorates toxicity of preconditioning to OPCs. OPCs derived from WT/KO SVZ spheres were treated either with or without preconditioning, and then exposed to lethal OGD 24 h later. (**A**) Double-labeled immunostaining of A2B5 and BNIP3. (**B**) Quantification of the percentage of BNIP3+ cells in the A2B5+ population. (**C**) Double-labeled immunostaining of A2B5 and AIF (blue, nuclei; green, A2B5 and red, AIF); (**D**) Quantification of the percentage of AIF nucleus translocation in the A2B5 + population. Results shown represent mean ± SD from 3 independent experiments. Bar, 10 μm.

We then explored the downstream mechanism of BNIP3-dependent OPC death in response to OGD. We performed immunocytochemical analysis to assess the expressions of BNIP3 (Figure 7A,B), and nucleus translocation of apoptosis-inducing factor (AIF) after OGD treatment in OPCs derived from SVZ spheres from both WT and BNIP3 KO adult mice (Figure 7C,D). Consistent with our Western blot data, lethal OGD alone or combined with preconditioning upregulated BNIP3 expression, which was concurrent with increased AIF translocation to the nucleus (Figure 7B,D). These results suggest that, under OGD conditions, BNIP3 stimulates OPC death by promoting AIF nucleus translocation, which was significantly decreased in OPCs derived from BNIP3 KO mice. Most importantly, when preconditioned OPCs were exposed to lethal OGD, BNIP3 deficiency completely blocked the toxicity caused by preconditioning. These data indicate that BNIP3 might be a major mechanism in acute and repeated hypoxic-ischemic injury to OPCs.

## 4. Discussion

In the present study, we have observed that cerebral ischemic preconditioning aggravates death of oligodendrocytes and identified that BNIP3 is involved in OPCs loss after preconditioning. The unfavorable effect of BNIP3 on OPCs adversely affected ischemic adaptation. Repeated ischemia increased OPCs vulnerability through stabilizing BNIP3 and promoting BNIP3-mitochondria binding. Knocking out of BNIP3 or blocking BNIP3 mitochondria integration with Nec-1 maximized the preconditioning protection of the brain. Therefore, BNIP3 inhibition should not be neglected as a complementary approach to preconditioning.

From experimental work done over several decades, neuroprotective therapies for ischemic stroke remain an interesting strategy to counter ischemic injury and suppress brain tissue damage. It’s still challenging because of the complexity of the stroke cascade. When different strategies have been implemented in randomized controlled trials, all of them failed to show a clinical benefit in patients with acute ischemic stroke. Mimicking the mechanisms of endogenous protection process is now a potential strategy for stroke prevention. Studies on non-pharmacological treatment strategies, especially preconditioning, emphasize the body’s naturally occurring neuroprotective mechanisms and may lead to new therapies and preventive measures for persons at high risk of ischemia and other neurological disorders. Ischemic preconditioning is a phenomenon that tissue is exposed to a brief, sublethal period of ischemia, which activates endogenous protective mechanisms, thereby reducing cellular injury that may be caused by subsequent lethal ischemic events [23]. Research on preconditioning over the past decade has resulted in various promising strategies, from experimental to clinical use [24,25]. Most preconditioning studies exclusively focus on the integrity of neurons, but detailed studies on the glial response (particularly OLs) after preconditioning are comparatively rare. Astrocytes have always been viewed as supporters of neuronal function due to increased glycogen storage, clear glutamate, and attenuate gap junction after preconditioning [26]. Microglia proliferation occurs in response to an unknown nonlethal injury to neurons or glia; this preconditioning-induced microglial proliferation has been suggested to be beneficial [27]. However, the impact of preconditioning on OPCs has not been well explored.

OPCs are typically identified by NG2 or PDGFRα expression, and they are the main source of mature myelinating OLs [28]. Compared to mature OLs, OPCs had a higher metabolic rate necessary for myelin initiation and repair. A stroke or even a tiny ischemic attack (consider as protective preconditioning) might be fetal to OPCs, which have a low tolerance for energy failure [29]. Compromised OPCs are presumably related to imperfect remyelination as well as scattered destruction of axons following cerebral ischemic injury. Enhancing the long-term survival of newly born and mature OLs contributes to improved white matter function at time points following cerebral ischemic injury. It’s demonstrated that small ischemic attacks as preconditioning stimuli may lead to functional impairment and behavioral deficits; furthermore, preconditioning necessarily involves some form of brain damage [30]. Therefore, we believe preconditioning needs to be reconsidered because the central belief is that “neuroprotective preconditioning” must be sub-threshold and should not cause damage to all the cell types in CNS [31].

Our earlier studies suggested that OPCs were highly sensitive to hypoxia, and short duration of ischemia stimuli was sufficient to exaggerate OPCs death in experimental stroke by loss of MCT1 [32,33]. As OPCs actively generate and export lactate into the extracellular space through bidirectional MCT1 in response to ischemia, our observations indicate that MCT1 is upregulated at the early stage of ischemia but turns downregulation while ischemia persisted. When the pH in OPCs is lower than 7.2, the lactate export is significantly inhibited, and intracellular lactate begins to accumulate rapidly. Such lactate export inhibition is detectable even in a mild ischemic condition equivalent to ischemic preconditioning [33]. We assumed that MCT1 loss in OPCs causes activation of the BNIP3 cell death pathway, potentially in concert with acidosis induced by lactate accumulation. It would be of interest to test this hypothesis to determine the consequences of MCT1 loss in OPCs at either short or repeated ischemia that is closely related to preconditioning.

BNIP3 (formerly known as NIP3) is a 194-amino acid pro-apoptotic BH3-only member of the Bcl-2 protein family. BNIP3 is apoptogenic and implicated in cell elimination in a variety of cells, including myocytes and neurons under ischemic conditions [21,34]. Under physiological conditions, the level of BNIP3 expression is low. BNIP3 is normally located in the cytosol and loosely attaches to the mitochondrial membrane. BNIP3 is overexpressed or induced following hypoxia or exposure to stressful stimuli. BNIP3 is one of the most upregulated genes after exposure to hypoxia by microarray analysis [35,36]. When BNIP3 is overexpressed, it inserts into the membrane of the mitochondria or the endoplasmic reticulum mediated by BNIP3′s TM domain, opens the permeability transition pore, and leads to loss of mitochondrial membrane potential, causing caspase-independent cell death [37]. BNIP3 is considered the upstream regulator of AIF and endonuclease G, which have previously been implicated in programmed necrosis [21,38]. It has been observed that BNIP3 can also cause autophagy, and the loss of BNIP3 protects against hypoxia-induced autophagic cell death [39]. However, these studies did not address the potential for BNIP3 to regulate OPCs’ sensitivity following ischemic injury in the brain. CNS exhibit different vulnerabilities to preconditioning, such that neurons, microglia, and astrocytes can trigger adaptation, yet OPCs could not be retained. The tolerance tends to be lower in OPCs, and failure to rescue OPCs would be the cause of imperfect myeline regeneration at the recovery stage following ischemic stroke. Our study clearly shows that inhibiting BNIP3 could help control OPCs apoptosis and thereby contribute to maintaining or modulating OPCs composition, as well as oligodendrocyte homeostasis. It’s conceivable that BNIP3 inhibition could be a complementary approach to improve the efficacy of preconditioning for ischemic stroke.

## Data Availability

The datasets used and analyzed during the current study are available from the corresponding author upon reasonable request.

## Supplementary Materials

The following supporting information can be downloaded at: https://www.mdpi.com/article/10.3390/biom12121872/s1, Figure S1: Preconditioning protocol screening.
