# Supplementary material for: Cerebral Ischemic Preconditioning Aggravates Death of Oligodendrocytes
Source: Biomolecules. 2022 Dec 14;12(12):1872. doi: 10.3390/biom12121872 (PMC9776065; doi:10.3390/biom12121872)
Supplement: Supplementary file 1 [file biomolecules-12-01872-s001.zip › biomolecules-2020708-supplementary.pdf]

Supplement Figure S1 Preconditioning protocol screening

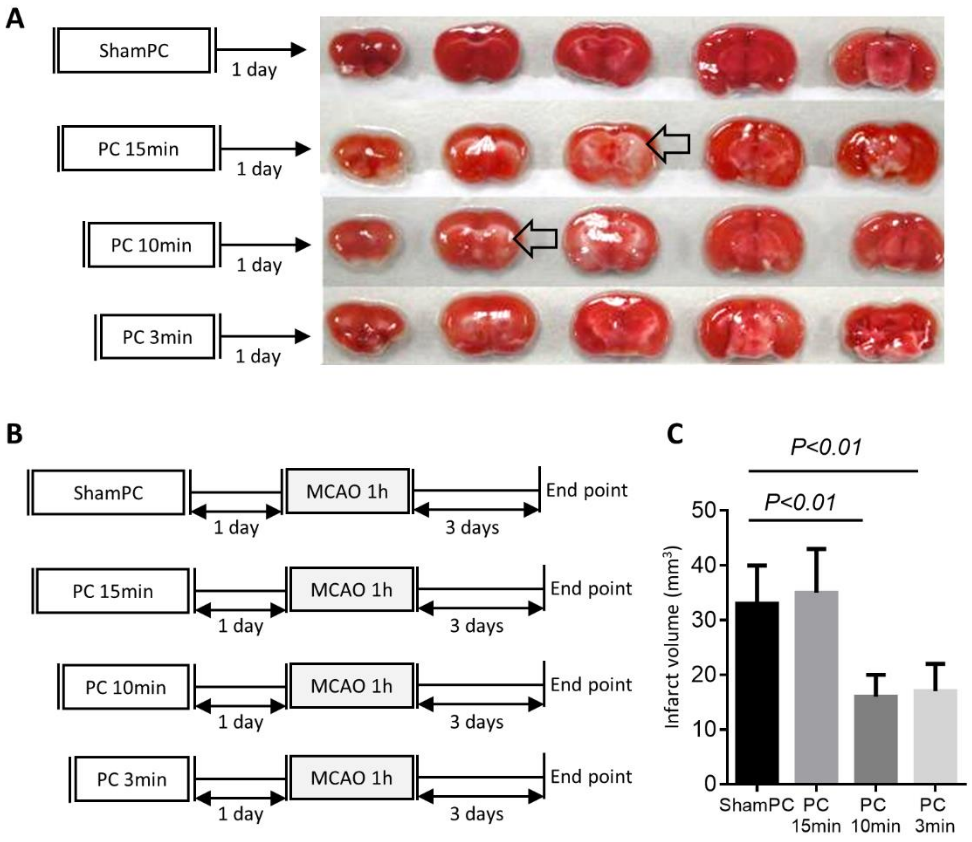

Supplement Figure S1 Preconditioning protocol screening. (A) The MCA was occluded for various periods (from 15 min to 3 min), and 1 day later, brain damage was assessed by TTC staining of coronal brain sections. Arrows showed striatal infarct. (B) As shown in the experimental protocols, mice received PC for various periods (from 15 min to 3 min) 1 day before 1-hour MCAO. Three days later, the result of infarct volumes was summarized in (C). Results shown represent mean±SD., n = 3.
